# A Double FBGs Temperature Self-Compensating Displacement Sensor and Its Application in Subway Monitoring

**DOI:** 10.3390/ma15196831

**Published:** 2022-10-01

**Authors:** Hongli Li, Gang Xu, Xin Gui, Lei Liang

**Affiliations:** 1 School of Mechanical Engineering, Hubei Engineering University, Xiaogan 432100, China; 2 School of Information Engineering, Wuhan University of Technology, Wuhan 430070, China; 3 National Engineering Research Center of Optical Fiber Sensing Technology and Networks, Wuhan University of Technology, Wuhan 430070, China

**Keywords:** displacement sensor, FBG, subway monitoring

## Abstract

In order to ensure the effective vibration–reduction and vibration–isolation of the steel spring floating plate rail and meet the safe operation requirements of the subway, a Fiber Bragg Grating (FBG) displacement sensor for the deformation monitoring of the subway floating plate is proposed. The sensor adopts double FBGs to realize temperature self-compensation. The elastic ring is used as the elastic conversion structure after the fiber grating is pre-stretched; the two ends are pasted and fixed in the groove in the diameter direction of the ring, which avoids the waveform distortion caused by the full pasting of the fiber grating. The combination of linear bearing and displacement probe rods can increase stability and reduce friction loss so that the sensor has the advantages of high sensitivity and accurate measurement results. The test results and error analysis show that in the range of 0~20 mm, the sensitivity of the sensor is 164.2 pm/mm, the accuracy reaches 0.09% F.S, and the repeatability error and hysteresis error are only 1.86% and 0.99%, respectively. The thermal displacement coupling experiment proves that the sensor has good temperature self-compensation performance. It provides a new technical scheme for the effective monitoring and condition assessment of the built-in steel spring floating plate rail.

## 1. Introduction

The subway provides great convenience for people’s travel and life. However, the vibration and noise pollution caused by subway operations have affected the residents and buildings along the line to varying degrees [1,2]. How to achieve effective vibration reduction and isolation has always been an environmental problem to be solved. The vibration and noise reduction measures are mainly to reduce the excitation intensity of the vibration source and cut off or weaken the vibration on the transmission path, such as in locomotive and vehicle, track, tunnel, foundation, ground surface, and surrounding buildings [3,4]. As an effective measure to reduce vibration and noise, steel spring floating slab track structures have been widely used all over the world. In order to achieve the long-term effect of vibration and noise reduction and meet the requirements for safe operation of subway, it is necessary to evaluate whether the built-in steel spring floating slab track meets the requirements. Therefore, it is of great significance to develop a monitoring sensor and system suitable for the floating slab track by using reasonable monitoring means for accurate and reliable diagnosis [5,6].

At present, more and more scholars have studied the vibration reduction and noise reduction of the track [7,8,9]. Some have studied the vibration reduction and isolation measures, some have studied the vibration reduction evaluation methods, and some have studied how to monitor the track status during subway operation. In terms of monitoring means, it is still relatively simple—mainly traditional electromagnetic sensors. Although such sensors are mature and relatively cheap, they have poor compatibility with the subway track. It is difficult to install a large number of sensors on the track, and the sensors are vulnerable to electromagnetic interference, affecting measurement accuracy and stability [10].

The Fiber Bragg grating (FBG) sensor has the natural advantages of anti-electromagnetic interference and can also use wavelength coding for detection [11,12]. A single fiber can be connected in series with multiple gratings of different wavelengths for network measurement. It has been widely used in the fields of structural health monitoring such as mechanical equipment [13], ocean engineering [14], ship [15], bridges [16], aerospace [17], robots [18], etc. In terms of subway monitoring, fiber Bragg gratings are also applied. Li’s team used a weak FBG array to conduct a series of studies on surface introduction event identification for subway tunnels, train tracking, intrusion detection, and track status monitoring [19,20,21,22]. Although this method can monitor many aspects of the subway, it requires a lot of data accumulation in the state judgment and the establishment of enough sample databases. The vertical displacement of the floating slab is one of the important parameters to evaluate whether the built-in steel spring floating slab track meets the specifications during the subway operation. The displacement measurement is a simple and effective method [23]. Although the existing FBG displacement sensors cannot be directly used for the measurement of floating plates (mainly reflected in the installation mode and measurement accuracy), there are many cases that can provide ideas for designers [24,25,26]. Rapid data transmission and fast signal processing are also important consideration indices of the monitoring system [27]. Fortunately, the fast demodulation and online data processing system of FBG is also mature, which can facilitate the integration of the floating plate condition monitoring system [28,29].

In this paper, combined with the actual engineering needs of the subway floating plate deformation measurement, an FBG displacement sensor is designed that can be installed by using the hollow structure of the floating plate vibration isolator. The displacement sensor adopts a double FBGs design, which can realize temperature compensation and improve sensitivity. The displacement sensor uses an elastic ring as the elastic conversion structure. After being pre-stretched, the two FBGs are installed in the ring groove perpendicular to each other. It is worth noting that only the two ends of the optical fiber are pasted with the ring, which avoids the waveform distortion caused by the full pasting of the fiber grating. The combination of the linear bearing and the displacement probe rod increases the stability and reduces the friction loss and also makes the sensor have the advantages of high sensitivity, long-term reciprocating measurement, and accurate measurement results. Finally, the prototype of the displacement is fabricated, and a comprehensive performance test is carried out, which further shows that the displacement sensor has a good measurement capability and a broad application prospect.

## 2. Sensor Structure and Measurement Principle

**Figure 1:**
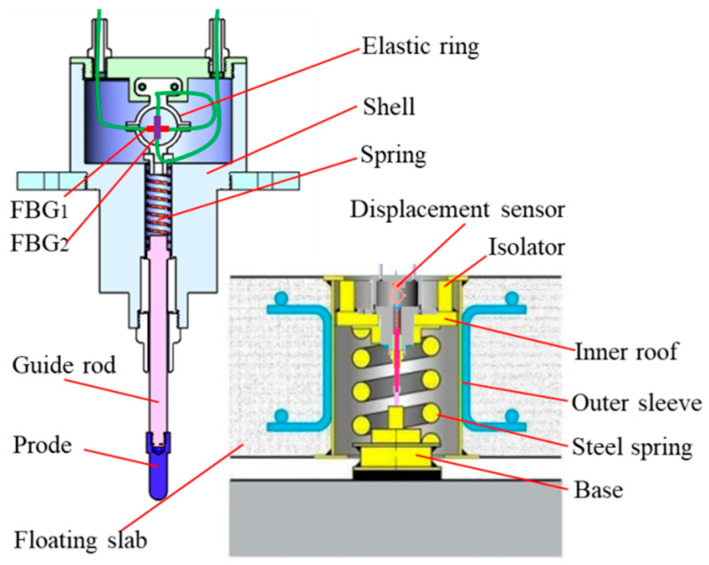
FBG displacement sensor and installation.

The steel spring vibration isolator is a kind of vibration isolation method used for the subway floating plate. This vibration isolator has a compact structure and there is no remaining space around to install the sensor. Therefore, we use the inner ring space of the steel spring to design a displacement sensor that can be installed internally. Although the temperature in the subway changes little (usually maintained between 25 °C and 35 °C), it is not a constant temperature. Fiber Bragg grating is sensitive to temperature and axial strain at the same time, so it is very necessary to compensate for the temperature of the sensor. The specific structure and installation method of the displacement sensor with temperature compensation is shown in Figure 1. The sensor shell is installed on the top plate of the vibration isolator, and the sensor probe is in contact with the bottom of the vibration isolator. FBG_1_ and FBG_2_ are installed on the elastic ring vertically and parallel to the displacement direction respectively. When the floating plate moves down, the vibration isolator is compressed, FBG_1_ wavelength increases, FBG_2_ wavelength decreases, and vice versa. By decoupling the wavelengths of the two FBGs, the displacement of the floating plate and the change in ambient temperature can be obtained.

**Figure 2:**
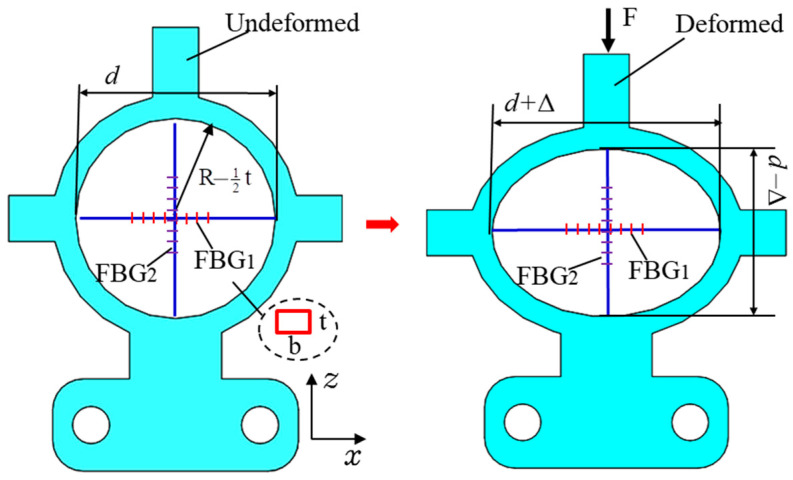
Stress diagram of elastic ring structure.

The FBG displacement sensor converts the measured displacement into the axial strain of the grating through the elastic conversion structure. By measuring the wavelength drift of the grating, the external measured displacement can be retrieved. The sensitive elastic body model is simplified to obtain the schematic diagram of the displacement sensing principle of elastic ring structure as shown in Figure 2.

Supposing Δ*L* is the external displacement of the probe, *F* is the elastic force of the spring, and Δ is the deformation of the elastic ring. According to the theory of small deformation [30], it can be considered that the deformation of the elastic ring in the direction of the intersecting axis is equal, and the deformation of the ring is obtained:

(1) $$\Delta =\frac{FR^{3}}{EI}(\frac{\pi }{4}-\frac{2}{\pi })$$

where, *R* is the average radius of the elastic ring; *E* is Young’s modulus of the cantilever beam; *I* is the moment of inertia of the section, whose value is $I=\frac{bt^{3}}{12}$, *b* is the width and *t* is the thickness.

According to FBG measurement theory, the relationship between the axial strain, temperature, and the wavelength drift can be obtained as follows [31]:

(2) $$\frac{\Delta \lambda_{B}}{\lambda_{B}}=(1-P_{e})\Delta \varepsilon +\left(\alpha +\zeta \right)\Delta T$$

where, $\lambda_{B}$ is the FBG center wavelength; $\Delta \lambda_{B}$ is the FBG wavelength drift; Δε is the axial strain change; Δ*T* represents the temperature increment; and *α*, *ξ*, and $P_{e}$ are the thermal expansion coefficient, the thermal–optic coefficient, and the elasto–optic coefficient (about 0.22 at room temperature), respectively.

When the external ambient temperature remains constant, the FBG wavelength change is only related to strain. If the stiffness coefficient of the spring in the displacement sensor is *k*, the relationship between FBG wavelength change and displacement can be expressed as:

(3) $$\frac{\Delta \lambda_{B}}{\Delta L}=K_{L}=\lambda_{B}\left(1-p_{e}\right)\left(\frac{\pi }{8}-\frac{1}{\pi }\right)\frac{kR^{2}}{EI}$$

Since the thermal expansion coefficient of the elastic ring material is greater than that of the optical fiber material, the relationship between FBG wavelength change and temperature is mainly affected by the elastic ring material, making its coefficient *K_T_*. In this paper, the two FBGs have opposite sensitivity to displacement and the same sensitivity to temperature, then:

(4) $$\left[\begin{array}{c} \Delta \lambda_{1}\\ \Delta \lambda_{2} \end{array}\right]=\left[\begin{array}{cc} K_{L} & K_{T}\\ -K_{L} & K_{T} \end{array}\right]\left[\begin{array}{c} \Delta L\\ \Delta T \end{array}\right]$$

That is, after conversion, we can get Δ*L*, Δ*T*, and displacement sensitivity *s_D_*:

(5) $$\left[\begin{array}{c} \Delta L\\ \Delta T \end{array}\right]=\left[\begin{array}{cc} \frac{1}{2K_{L}} & -\frac{1}{2K_{L}}\\ \frac{1}{2K_{T}} & \frac{1}{2K_{T}} \end{array}\right]\left[\begin{array}{c} \Delta \lambda_{1}\\ \Delta \lambda_{2} \end{array}\right]$$

(6) $$s_{D}=\frac{\Delta \lambda_{1}-\Delta \lambda_{2}}{\Delta L}=2K_{L}$$

In the proposed sensor, the spring is made of manganese steel, and its stiffness coefficient is 1.28 N/mm. The elastic ring is made of stainless steel with an elastic modulus of 2.06 × 10^11^ Pa. The general effective length of fiber Bragg grating is about 10 mm, in order to avoid sticking FBG to the elastic ring when FBG fiber is stuck in the elastic ring, which will affect the measurement effect, the inner diameter of the elastic ring can be appropriately increased to leave error for fiber sticking, so the inner diameter *d* of the elastic ring is set to 13 mm, which is slightly larger than the effective length of fiber Bragg grating. Therefore, the average radius of the elastic ring *R* is equal to 6.5 + 0.5 *t*. 

**Figure 3:**
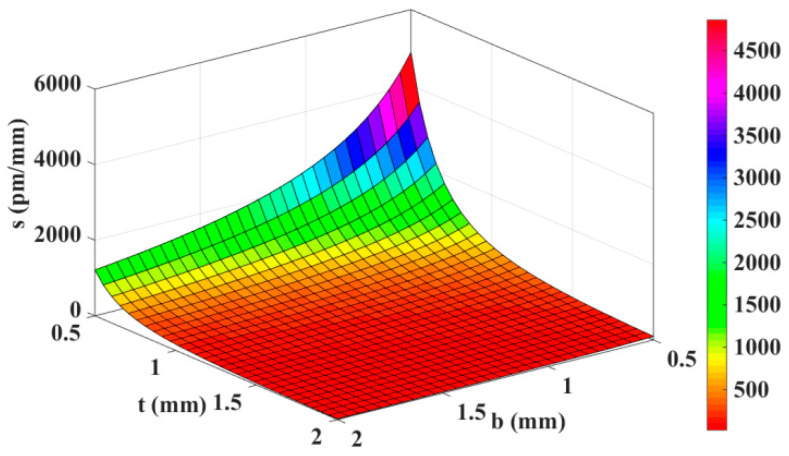
Relationship between sensitivity and elastic ring size.

In order to ensure the excellent sensing performance of the sensor, it is necessary to optimize the size of the thin-walled ring. Next, the relationship between the sensor sensitivity *s* and the width *b* and thickness *t* is given, as shown in Figure 3.

Considering the actual situation of fiber Bragg grating in general engineering projects, the FBG wavelength change generally does not exceed 3 nm, and a certain amount of pre-stretching of optical fiber (usually about 1 nm) is required during the preparation of the sensor. When preparing the sensor, the FBG demodulator is connected to observe the FBG wavelength, and the pre-stretched length is controlled by gradually increasing the weight mass. The purpose of pre-stretching is to keep the FBG fiber in the pre-stretching state when the sensor is working to prevent the FBG from becoming relaxed due to the influence of the temperature environment, thus affecting the displacement measurement. Therefore, for the sensor in this paper, when in use, FBG_1_ is stretched and the wavelength shift should preferably be within 2 nm under the full scale. Moreover, FBG_2_ is compressed, and its pre-stretching wavelength should be greater than 2 nm. Therefore, taking the width of the elastic ring *b* = 1.2 mm and the thickness *t* = 1.5 mm and according to the above formula, the wavelength change of FBG_1_ and FBG_2_ is 1.732 nm and −1.732 nm at the full scale (L = 20 mm), respectively, the sensitivity of the displacement sensor *s* is calculated to be 173.2 pm/mm. In addition, in order to firmly fix the FBG fiber on the ring structure, 353 nd adhesive produced by EPO-TEK is used for curing at 120 °C for 15 min and then natural cooling.

## 3. Performance Test of Displacement Sensor

To ensure that the proposed FBG displacement sensor can work reliably in the harsh field environment of the subway for a long time, it is necessary that a comprehensive performance test is conducted. First, all the assembled FBG displacement sensors are put into the incubator for an aging test at 20 °C~80 °C, which is kept at each temperature node for one hour with a step size of 10 °C, and heating and cooling are repeated ten times. Then, a series of performance tests are carried out, mainly including sensitivity experiments and temperature characteristic experiments, and error analysis is carried out.

### 3.1. Sensitivity Calibration Experiment

Owing to any sensor that has been designed, processed, and assembled with different sensing characteristics, all FBG displacement sensors designed with the same structure need to be calibrated before they are put into use. Sensitivity is one of the important indexes of the sensor.

**Figure 4:**
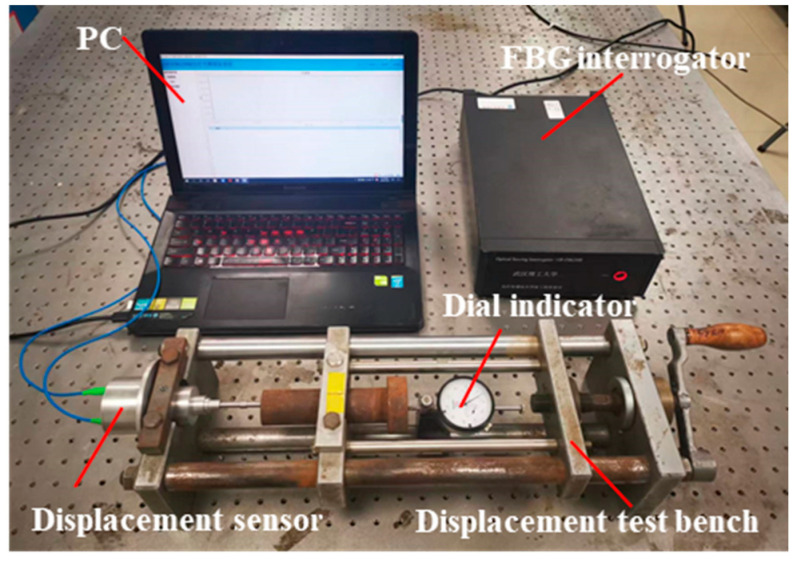
Displacement calibration experimental device.

The calibration experiment of sensitivity is carried out in the laboratory with constant temperature, as shown in Figure 4. The FBG displacement sensor and dial indicator (accuracy: 0.01 mm) are fixed on the test bench respectively. The FBG sensor probe and dial indicator probe are in contact with the sliding block of the test bench respectively, and the movement of the sliding block can drive the FBG sensor and indicator probe to produce displacement. The FBG sensor is connected with the FBG interrogator (accuracy: 3 pm; resolution: 0.1 pm) through a fiber optic cable to receive and record the change of reflected wavelength under the action of external displacement.

The operation steps of the calibration experiment are shown below: First, the calibration was performed from 0 to full range (20 mm) with 5 mm as the step, holding for 3~5 s at each displacement node, respectively, and waiting for the FBG wavelength to stabilize, and then unloading the displacement back to the initial position (that is the zero position) for the same interval. The displacement loading and unloading test process is repeated three times. Last, the real-time data of the experiment are collected at the acquisition frequency of 100 Hz by the fiber grating demodulator. 

**Figure 5:**
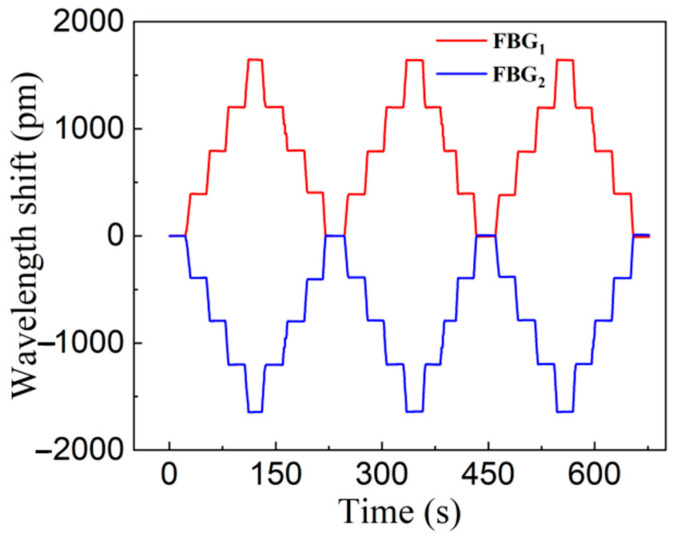
Wavelength shift versus time in three periods of displacement cycle test.

**Figure 6:**
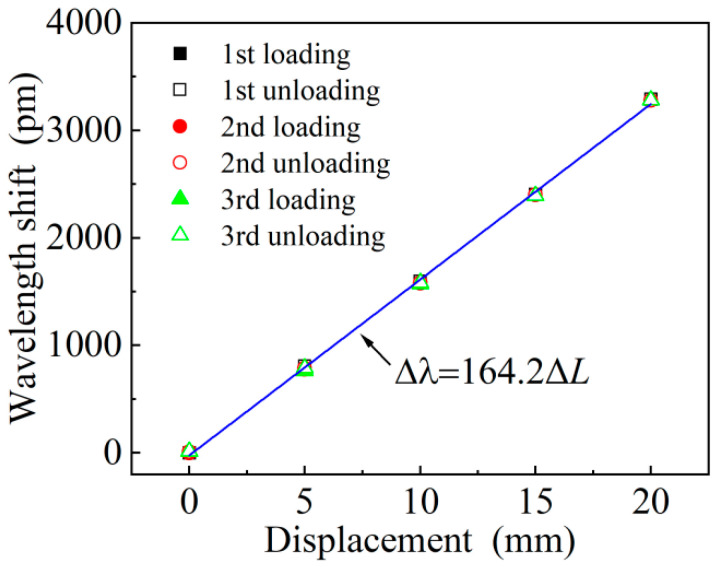
Wavelength shift versus displacement during three periods of displacement cycle test.

Figure 5 shows the time history diagram of FBG_1_ and FBG_2_ wavelength shift for three displacement cycle tests, and the wavelength drift data when the displacement loaded and unloaded at each displacement node are obtained, and the six displacement cycle test data diagrams are obtained shown in Figure 6. The six displacement curves are then arithmetically averaged, and then by linear fitting, the theoretical relationship of the difference between the displacement and the wavelength drift is obtained.

According to the displacement fitting curve in Figure 6, Δλ=164.2ΔL, R=0.999. It can be seen that the FBG displacement sensor has good linearity and its displacement sensitivity $s_{D}$ is 164.2 pm/mm. According to the long-term test of the FBG interrogator, the demodulation accuracy $I_{a}$ is about 3 pm and the resolution $I_{r}$ is 0.1 pm. Therefore, the displacement measurement accuracy of the sensor is $\frac{I_{a}}{s_{D}\cdot \Delta L}=0.09\%\;F.S$ and the resolution is $\frac{I_{r}}{s_{D}}=6.1\times 10^{-4}\;$mm. In addition, according to the data of three cycle tests, the maximum repeatability error of the six curves is calculated at the displacement of 5 mm, and its value $e_{R}$ is ησ/ΔL=1.86% (*η* = 3, which is the coverage factor; *σ* is the standard deviation, $\sigma =\sqrt{\frac{1}{2N}\left(\sum_{i=1}^{N}\sigma_{U_{i}}^{2}+\sum_{i=1}^{N}\sigma_{D_{i}}^{2}\right)}=0.124$). In the three process return cycles, the maximum hysteresis occurs at 5 mm of the second cycle, the hysteresis is 32.8 pm, and the hysteresis error $e_{H}$ is calculated as $e_{H}=\Delta H_{\max}/\Delta_{FS}=0.99\%$. The results show that the designed displacement sensor can meet the engineering needs of high precision.

According to the experimental test results, the sensitivity of the sensor is 164.2 pm/mm, which is slightly different from the theoretical value of 173.2 pm/mm. This is mainly due to the machining accuracy and assembly accuracy error of the deformation ring and the FBG pasting process.

### 3.2. Temperature Characteristic Test

**Figure 7:**
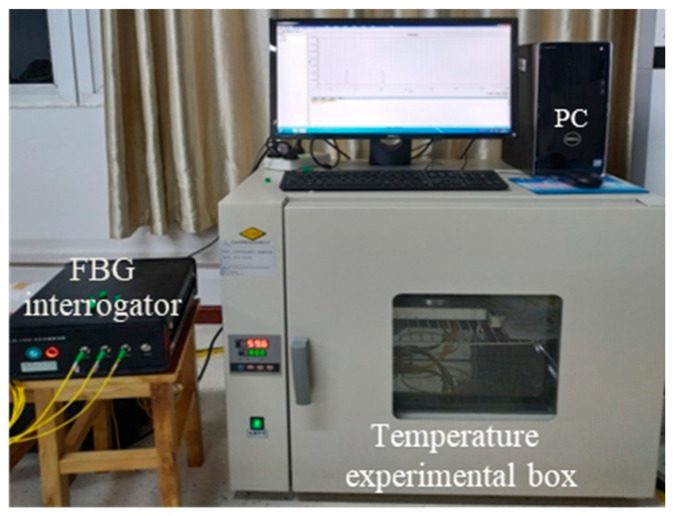
Temperature test device.

**Figure 8:**
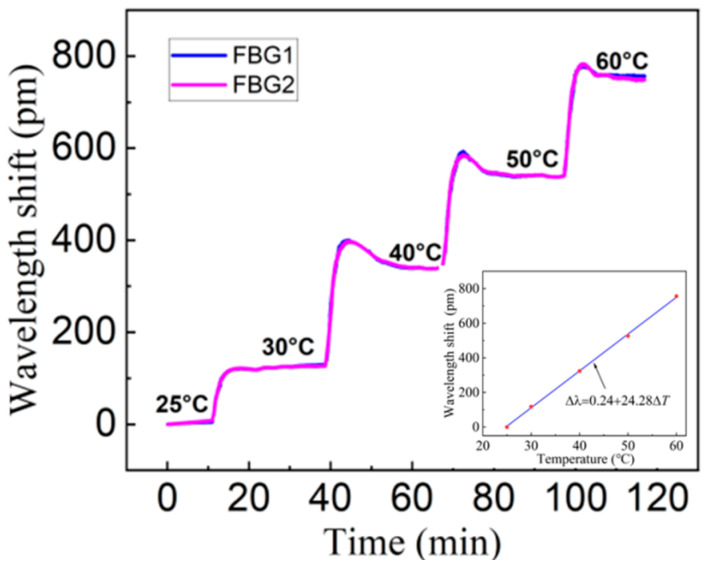
Wavelength shift versus time curves in the temperature compensation.

The wavelength shift of FBG is affected by both axial strain and temperature at the same time. In order to verify the temperature compensation ability of the sensor designed in this paper, the temperature characteristics of the sensor are tested. Figure 7 shows the experimental device diagram of temperature compensation characteristics. One sensor is randomly selected and placed in the temperature experimental box (resolution: 0.5 °C; accuracy: 1 °C), and the FBG interrogator collects the wavelength in real-time at the frequency of 20 Hz. The temperature experimental box is produced by Hefei Kejing Material Technology Co., Ltd. (Hefei, China), the model is OTF-1200X, the temperature range is −20~130 °C, the resolution is 0.5 °C, the accuracy is 1 °C, and the working room size is 40 mm × 50 mm × 40 mm. Figure 8 shows the time–history curve of the sensor wavelength changing with temperature (25 to 60 °C). It can be seen that two FBGs have almost identical temperature curves. The temperature sensitivity of a single FBG is 24.28 pm/°C, which is mainly related to the thermal expansion coefficient of the elastomer material. The sensitivity curve can provide the basis for the temperature compensation of the sensor.

### 3.3. Thermal-Displacement Coupling Test

**Figure 9:**
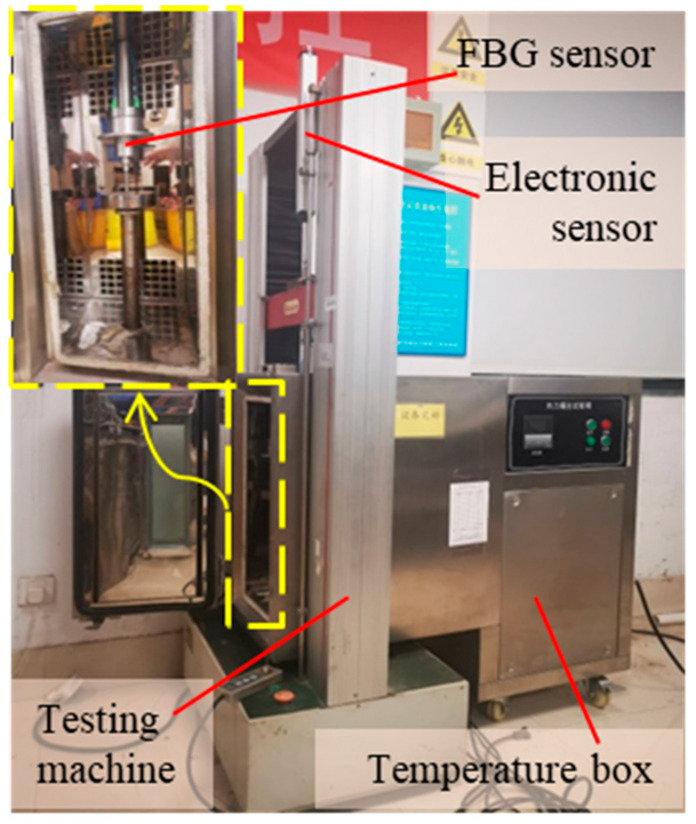
Thermal–displacement coupling test device.

In order to better verify the temperature self-compensation ability of the sensor designed in this paper, the author carried out a thermal displacement coupling test, that is, the test is carried out when the temperature and displacement change at the same time. As shown in Figure 9, the FBG sensor is placed on the static stretching machine with a temperature control box, and the electronic displacement sensor is placed outside the temperature control box as a reference sensor to eliminate the influence of temperature change on the electronic displacement sensor.

**Figure 10:**
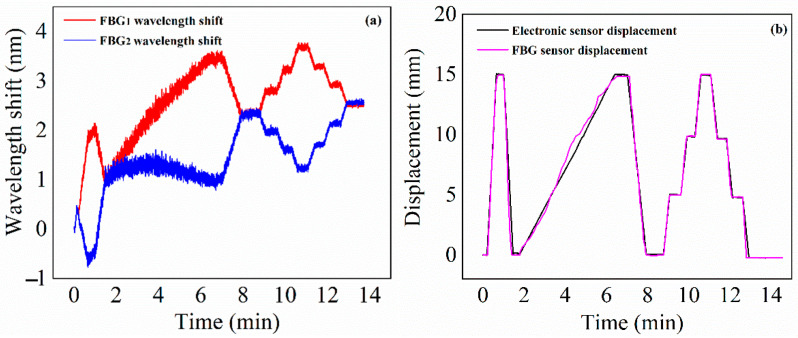
(**a**) FBG wavelength shift with displacement and temperature; (**b**) displacement comparison curve of FBG sensor and electronic sensor.

The testing machine reciprocates in the range of about 0–15 mm at different speeds. At the same time, the temperature control box is raised by 10 °C from the room temperature at a rate of about 1 °C/min. The test results are shown in Figure 10. Figure 10a is the wavelength shift of FBG_1_ and FBG_2_ with displacement and temperature, and Figure 10b is the comparison between the FBG sensor displacement after data processing and the electronic sensor displacement. It can be seen that, due to the influence of temperature, the symmetry of FBG_1_ and FBG_2_ wavelength shift is not good, but after data processing, the displacement curve of the FBG sensor is in good agreement with that of the electronic displacement sensor. The error is mainly caused by the large cavity of the temperature control box, the temperature in the cavity is not uniform, and the temperature received by the sensor has certain randomness. The displacement transmission rod and other fixtures in the temperature control box are also affected by temperature, which is another cause of measurement error. Through the above experiments, it is further proved that the FBG sensor has the ability for temperature self-compensation.

## 4. Results and Analysis

### 4.1. Static Load Test

**Figure 11:**
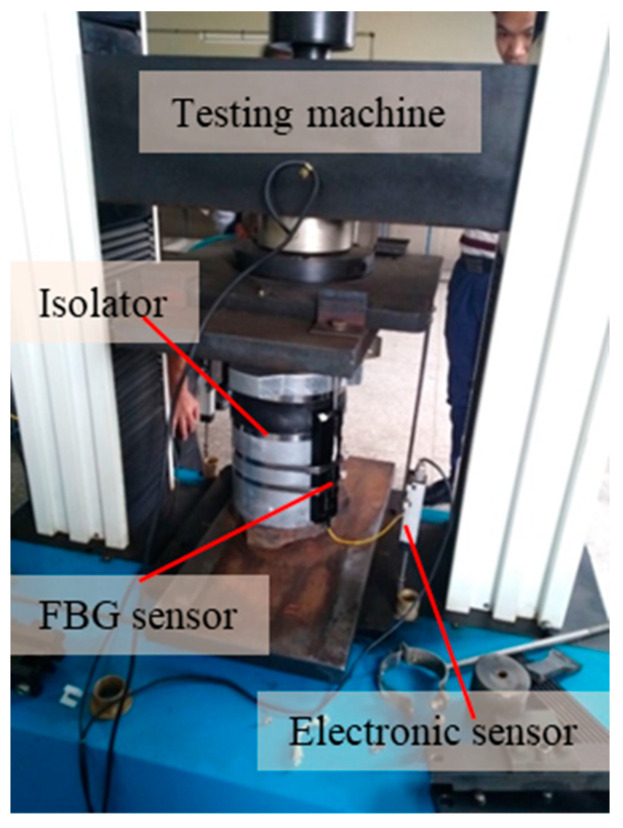
Static loading experiment of sensor.

Figure 11 shows the static load test site diagram of the sensor. The developed FBG displacement sensor is installed in the vibration isolator and measures the data on the testing machine, and the measured result is compared with the results of the electronic displacement sensor. The testing machine is ZCW-150 electronic universal testing machine produced by Jinan Zhongchuang Co., Ltd. (Jinan, China), the test force measurement range is 0.6 kN~150 kN, and the accuracy grade is 0.5. The electronic displacement sensor is a KTR12 linear displacement sensor of Shenzhen Miran Company (Shenzhen, China), with a range of 100 mm and a repeatability accuracy of 0.01 mm, meeting the test requirements of this experiment.

**Figure 12:**
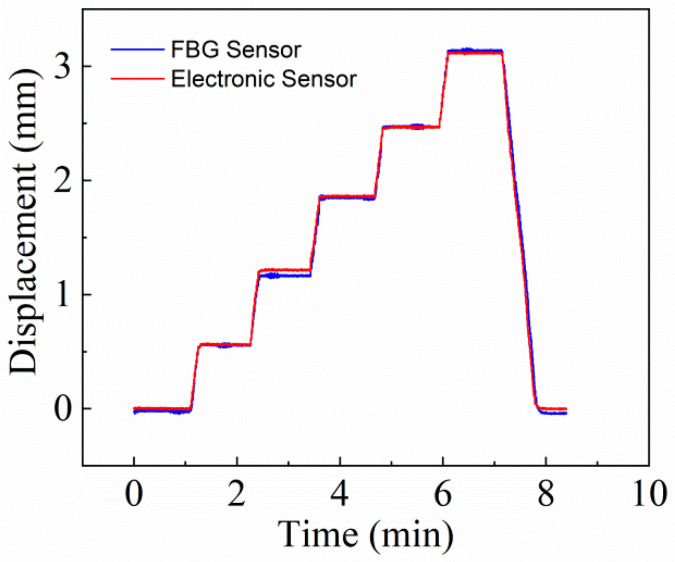
Comparison results of FBG sensor and electronic sensor.

The vibration isolator selected in this experiment is a special vibration isolator suitable for B-type subway trains with floating plate strength of C30, and its stiffness is 8 kN/mm. According to the usage specification of the floating plate, its maximum displacement does not exceed 3 mm, so the load is applied to the static testing machine from 0 to 25 kN in 5 kN steps. The test results are shown in Figure 12. It can be seen from the figure that the change trends of the FBG displacement sensor and the electronic displacement sensor are almost exactly the same, which also proves that the measurement results of the FBG displacement sensor are reliable.

### 4.2. Random Dynamic Load Tests

**Figure 13:**
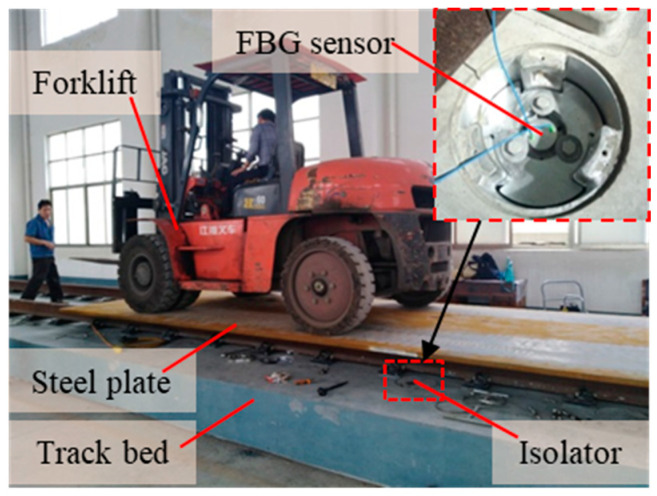
Random dynamic load test of sensor.

Ref. [30] has proved that the sensor with this structure can work continuously for 555 h at a frequency of 2.5 Hz, and the number of cycles reaches 5 × 10^6^, it can be seen that the sensor has good dynamic performance and is durable, working for a long time under periodic load. To verify the dynamic performance of the sensor under irregular loads, the tests are conducted on the indoor floating slab track bed (equipped with 16 vibration isolators). The forklift (weighing about 8.5 tons) is used as the loading tool. In order to facilitate the forklift to run on the track, it is necessary that a steel plate is placed on the track, and then the forklift is driven into the track. The experimental device is shown in Figure 13.

**Figure 14:**
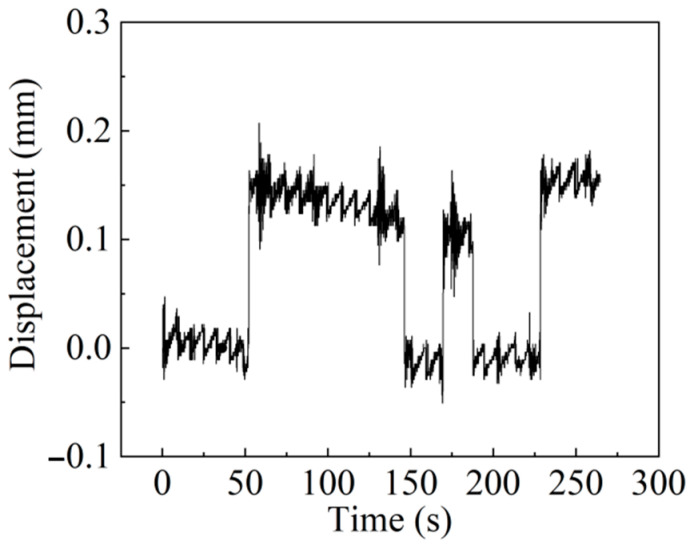
Sensor dynamic test results.

Figure 14 shows the displacement change of the sensor when the forklift drives on the track bed at different speeds three times. Due to the indoor test, there are only two track beds, which are short in length. The forklift cannot travel quickly, and the speed is basically controlled at 5~8 km/h. Of course, it is difficult to travel at a constant speed. The state of the steel plate placed is zero displacement state to start recording data. The forklift makes the floating plate displace about 0.15 mm. When the forklift drives on or out of the floating plate, the sensor signals have obvious steps. Due to the position change of the forklift on the floating plate and the vibration impact during driving, the floating plate will vibrate to some extent. Although the displacement is small, it is clearly reflected in the sensor signal, and the displacement change is about ±0.05 mm. It can be seen that the sensor also has good dynamic performance. This experiment is only to measure the dynamic performance of the sensor under random load, so only one displacement sensor is installed. Although it cannot measure the deformation of the whole floating plate, it can prove that the sensor itself has good performance. We will continue to pay attention to this work in the follow-up research and conduct a more comprehensive test.

## 5. Conclusions

According to the monitoring requirements of the subway floating plate, the maximum allowable displacement of the steel spring vibration isolator used in the subway floating plate is generally not more than 4 mm, which requires the monitoring sensor to have a high displacement accuracy, which is usually required to reach 1%, that is, 0.04 mm. In this paper, a dual FBGs displacement sensor with temperature compensation built into the vibration isolator was developed. By optimizing the structure of the elastic ring (the width *b* = 1.2 mm and the thickness *t* = 1.5 mm), the sensor has higher sensitivity and accuracy compared with previous work (ref. [30], sensitivity 36.36 pm/mm and accuracy 0.0825 mm). The calibration results and error analysis of the sensor showed that the sensor had a sensitivity of 164.2 pm/mm and an accuracy of 0.09% F.S (0.018 mm) in the range of 0~20 mm. The repeatability error and hysteresis errors were only 1.86% and 0.99%, respectively. Further, the thermal displacement coupling experiment proves that the sensor has good temperature self-compensation performance. Through the comparison with the electronic displacement sensor and the test under the random load of the forklift, it is proved that the sensor has good static and dynamic performance and can meet the test requirements of the steel spring isolator in the subway floating plate. According to the field measurement data, it was verified the feasibility of the developed FBG displacement sensor, which provided an effective detection and monitoring method for the evaluation of technical parameters of subway floating plate products and the safety early warning of engineering sites.
